# Retraction

**DOI:** 10.1111/jcmm.17181

**Published:** 2022-01-17

Long noncoding RNA MALAT1 enhances the docetaxel resistance of prostate cancer cells via miR‐145‐5p‐mediated regulation of AKAP12. Xue, D., Lu, H., Xu, H‐Y., Zhou, C‐X., He, X‐Z. *J Cell Mol Med*. 2018; 22: 3223–3237. https://doi.org/10.1111/jcmm.13604

The above article, published online on 06 April 2018 on Wiley Online Library (wileyonlinelibrary.com), has been retracted by agreement between the journal's Editor‐in‐Chief, Stefan N. Constantinescu, the authors, the Foundation for Cellular and Molecular Medicine, and John Wiley & Sons Ltd. The retraction has been agreed following concerns raised by the authors. In subsequent experiments, using newly‐sourced and properly validated PC3 cells from a different vendor, the authors were unable to reproduce the original findings of the wound healing assays. After careful analysis, they realized that the PC3 cells used in the original experiments were likely contaminated, which might have affected the results, and thus, the conclusions of the study.

