# Correction to ‘Retraction’

**DOI:** 10.1111/jcmm.18250

**Published:** 2024-03-20

Retraction. J Cell Mol Med, 26: 956‐956. https://doi.org/10.1111/jcmm.17181.

**Retraction:** ‘Long noncoding RNA MALAT1 enhances the docetaxel resistance of prostate cancer cells via miR‐145‐5p‐mediated regulation of AKAP12’” Xue, D., Lu, H., Xu, H‐Y., Zhou, C‐X., He, X‐Z. *J Cell Mol Med*. 2018; 22: 3223–3237. https://doi.org/10.1111/jcmm.13604. The above article, published online on 06 April 2018 on Wiley Online Library (wileyonlinelibrary.com), has been retracted by agreement between the journal's Editor‐in‐Chief, Stefan N. Constantinescu, the authors, the Foundation for Cellular and Molecular Medicine and John Wiley & Sons Ltd. The retraction has been agreed following concerns raised by the authors. In subsequent experiments, using newly sourced and properly validated PC3 cells from a different vendor, the authors were unable to reproduce the original findings of the wound healing assays. After careful analysis, they realized that the PC3 cells used in the original experiments were likely contaminated, which might have affected the results, and thus, the conclusions of the study.

Following the publication of the retraction notice above, additional concerns were raised by a third party regarding inappropriate duplications of images within the original article (https://doi.org/10.1111/jcmm.13604) as well as between the original article and other articles published previously elsewhere. An internal investigation into the concerns confirmed these allegations. Therefore, the editors have decided to issue a correction to the previously published retraction wording.

Please find the corrected retraction text as follows:

**Retraction:** Long noncoding RNA MALAT1 enhances the docetaxel resistance of prostate cancer cells via miR‐145‐5p‐mediated regulation of AKAP12. *J Cell Mol Med*. 2018; 22: 3223–3237. https://doi.org/10.1111/jcmm.13604. The above article, published online on 06 April 2018 on Wiley Online Library (wileyonlinelibrary.com), has been retracted by agreement between the journal's Editor‐in‐Chief, Stefan N. Constantinescu, the authors, the Foundation for Cellular and Molecular Medicine and John Wiley & Sons Ltd. The retraction has been agreed following concerns raised by the authors. In subsequent experiments, using newly sourced and properly validated PC3 cells from a different vendor, the authors were unable to reproduce the original findings of the wound healing assays. After careful analysis, they realized that the PC3 cells used in the original experiments were likely contaminated, which might have affected the results, and thus, the conclusions of the study. Subsequently, an investigation into concerns raised by a third party, revealed inappropriate duplication of images within the article (Figure 2C, 4E, 6E and 6C subpanels) as well as between this (Figure 5D, 7A and 7E) and other articles that were either previously published or published later in the same year in a different scientific context. Thus, the editors consider the conclusions of this manuscript substantially compromised.

